# Could Practices of Remembrance Reduce Social Isolation in Late Life? Ethnographic Evidence From Poland and Detroit

**DOI:** 10.1093/geroni/igaa057.2313

**Published:** 2020-12-16

**Authors:** Jessica Robbins

**Affiliations:** Wayne State University, Detroit, Michigan, United States

## Abstract

Because social isolation can have negative effects on older adults’ wellbeing, programs that reduce social isolation have potential to improve older adults’ wellbeing. One presumed aspect of these programs’ significance is the social connection occurring through the programs themselves. However, drawing on ethnographic data collected in Poland and Detroit, this presentation argues that practices of remembrance, in which older adults connect with deceased kin and loved ones, may offer possibilities for reducing social isolation. In Poland, older adults engage in practices of storytelling in which they remember deceased kin and lost homes and homelands. In Detroit, Michigan, older African Americans who garden remember their deceased kin and friends through the practice of gardening itself. This presentation presents a cross-cultural analysis of how older adults’ practices of remembrance may offer opportunities to reduce social isolation—even for older adults who live alone—by connecting to meaningful relations, times, and places.

